# A Lightweight Net with Dual-Path Feature Enhancer and Bidirectional Gated Fusion for Cloud Detection

**DOI:** 10.3390/s26051727

**Published:** 2026-03-09

**Authors:** Yan Mo, Puhui Chen, Shaowei Bai, Erbao Xiao

**Affiliations:** 1 College of Aeronautics Engineering, Nanjing University of Aeronautics and Astronautics, Nanjing 210016, China; 2 School of Information Engineering, Nanchang Hangkong University, Nanchang 330063, China; 2404085404317@stu.nchu.edu.cn (S.B.); 2504085401012@stu.nchu.edu.cn (E.X.); 3 State Key Laboratory of Mechanics and Control of Mechanical Structures, Nanjing University of Aeronautics and Astronautics, Nanjing 210016, China; phchen@nuaa.edu.cn

**Keywords:** cloud detection, lightweight network, dual-path feature enhancer, bidirectional gated fusion

## Abstract

Cloud detection serves as a critical preprocessing step in remote sensing image processing and quantitative applications. However, prevailing deep learning-based models often depend on computationally intensive backbone networks to achieve high accuracy, which hinders their deployment in resource-constrained scenarios such as on-board processing or edge computing. To bridge the trade-off between accuracy and efficiency, this paper introduces a lightweight network for cloud detection. The core innovations of our network are twofold: (1) a dual-path feature enhancer that operates at the front end to extract and fuse multi-scale features through a parallel architecture, significantly enriching feature diversity and representational capacity, thereby alleviating the need for a complex backbone, and (2) a bidirectional gated fusion module, which adaptively integrates multi-scale features from the dual-path feature enhancer with deep semantic features from the backbone decoder through a gated attention mechanism and dynamic convolution, thereby enhancing feature discriminability. Comprehensive experiments on the public HRC_WHU dataset demonstrate that the proposed model achieves a high overall accuracy of 96.31% and a mean intersection-over-union of 92.82%, with only 12.04 GFLOPs of computational cost, outperforming several state-of-the-art methods. These results validate that our approach effectively balances high detection performance with computational efficiency, offering a practical solution for real-time, lightweight cloud detection in high-resolution remote sensing imagery.

## 1. Introduction

With the rapid advancement of satellite remote sensing technology, high-resolution remote sensing images have found widespread application across numerous fields. However, cloud cover has posed a formidable challenge in remote sensing image processing, as it impedes the capture of surface information by optical sensors, resulting in data loss and potential biases in subsequent applications [1]. Consequently, cloud detection has remained a pivotal and challenging research focus in the preprocessing of remote sensing images within the remote sensing community.

Convolutional Neural Networks (CNNs) [2], leveraging local receptive fields, weight sharing, translation invariance, and pooling, excel in extracting spectral and geospatial features, enabling deep architectures for capturing high-level semantic representations crucial for cloud detection in remote sensing. DCNet [3] integrates deformable convolutions within the encoder–decoder framework to adaptively capture cloud morphology, addressing spatial information loss. The encoder–decoder architecture, however, faces issues of spatial information loss and feature dilution, prompting a shift in cloud detection methods from purely convolutional structures towards global feature extraction techniques, such as attention modules. Several studies [4,5] have begun to incorporate attention mechanisms into UNet [6]. CDUNet [7] incorporates Spatial Prior Self-Attention (SPSA) alongside a dual attention mechanism, which effectively reduces feature redundancy and enhances the network’s ability to detect clouds. CDnetV2 [8] and CFCA-Net [9] further leverage channel and spatial attention modules to refine feature maps and highlight critical color, texture, and spatial cloud features. Zhang et al.’s proposed cloud detection model [10] combines probabilistic upsampling with attention mechanisms to enhance cloud regions, while SEUNet++ [11] and AFMUNet [12] integrate lightweight channel/spatial attention modules for adaptive field of view adjustment, improving cloud detection performance.

To enhance deep learning networks’ ability to capture intricate features, researchers have explored multi-feature fusion strategies. Zhang et al. introduced CSD-Net [13], incorporating Multi-scale Feature Fusion (MFF) and Controllable Depth Supervision and Feature Fusion (CDSFF), effectively capturing salient semantic features for clouds and snow. Wang et al. designed ABNet [14] with an All-scale Feature Fusion (AF) module, enabling decoders to integrate features from all resolutions. GCDB-UNet [15] augmented UNet with Global Context Dense Blocks, boosting thin cloud detection. Wu et al.’s boundary-based model [16] strengthened multi-scale feature extraction for clouds of varying sizes. CRSNet [17] employed a Multi-scale Global Attention module, improving channel and spatial information for higher accuracy. BABFNet [18] used a boundary prediction branch to enhance cloud detection in complex regions. GANet [19] introduced GFAM and PAPM, bridging spatial details and high-level semantics while extracting multi-scale global features. MCDNet [20] leveraged MSFF to compensate for spatial information loss, improving sensitivity to fragmented clouds. Furthermore, augmenting feature inputs [21,22,23,24,25] demonstrates the effectiveness of multi-feature fusion in enriching network capabilities for cloud detection.

CNNs excel in local connections but struggle with global context capture, whereas Transformers, exemplified by ViT [26] and Swin Transformer [27], excel at global feature extraction and context understanding. Li et al. proposed a novel CS (Cloud and shadow) detection algorithm, CSDFormer [28], specifically utilizing a hierarchical converter structure in the encoder stage to extract CS features. Each converter layer incorporates multiple multi-head self-attention mechanisms for computing the long-range connectivity of pixels. Several studies combine Transformers with CNNs to enhance cloud detection networks. Lu et al. [29] and Gong et al. [30] integrate Swin Transformer and CNNs for semantic and spatial detail extraction. CNN-TransNet [31] employs a hybrid CNN-Transformer with Differential Feature Enhancement for improved cloud discrimination. Gu et al.’s [32] hybrid model incorporates Axial Shared Hybrid Attention and an Attention Guidance Module for efficient fusion. CD-CTFM [33] utilizes a lightweight CNN-Transformer encoder–decoder with attention gates. MAFNet [34] collaborates ResNet50 and Swin Transformer with Multi-branch Attention Fusion. The MMA network [35] leverages multi-scale overlapping blocks, PVT, strip convolution, and Multi-scale Global Aggregation for rich semantic extraction. These hybrid approaches demonstrate the potential of combining CNNs and Transformers for advanced cloud detection. Xu et al. proposed an Integration Transformer with gradient-aware feature aggregation (TransGA-Net) [36]. This framework employs Transformers as encoders, significantly enhancing the modeling capability for global features and long-term dependencies. Gao et al. proposed SwinCloud [37] for cloud detection in the thermal infrared spectral range. The network augments the Swin Transformer’s window attention module with a CNN-based parallel pathway to effectively model global-local information.Zhou et al. proposed a dual-branch collaborative optimization network (DFC-Net) [38], which achieved cross-scale feature fusion and detail enhancement through a dual-branch interaction mechanism that combines global context modeling and local detail perception.

However, state-of-the-art cloud detection models predominantly rely on computationally intensive backbone networks, leading to substantial parameter counts and slow inference speeds. This hinders their deployment on resource-constrained platforms such as edge devices or satellite-borne systems. Existing approaches are often caught in a trade-off: they either enhance detection accuracy by increasing network complexity—sacrificing practicality—or prioritize efficiency through excessive architectural simplification. This simplification particularly compromises the model’s ability to represent weak features (e.g., thin clouds) and precise boundaries, which are critical in challenging scenarios like urban landscapes and snow-cloud coexistence, thereby fundamentally limiting detection performance under these conditions.

To address these limitations, this paper introduces a lightweight cloud detection network. The proposed model comprises four core components: a dual-path feature enhancer, a lightweight backbone network, a progressive feature pyramid decoder, and a bidirectional gated fusion module. Through this systematic and lightweight-oriented design, the network maintains competitive detection accuracy while significantly improving computational efficiency. The main contributions of this work are summarized as follows:

- We propose a dual-path feature enhancer, deployed at the front end of the network. It consists of two dedicated paths that respectively capture global structural information and local detail features. An adaptive fusion mechanism is introduced to effectively integrate multi-scale representations from both paths. This design considerably enriches the expressiveness of input features and reduces reliance on a heavy backbone network.
- We design a bidirectional gated fusion module. It employs a bidirectional gated attention mechanism to adaptively select informative features from both the multi-scale feature stream (provided by the front-end enhancer) and the deep semantic stream (from the backbone decoder). Combined with dynamic convolution and an enhanced attention mechanism, the module establishes a “selection–fusion–refinement” workflow that preserves spatial details and strengthens semantic consistency during feature fusion.

Experimental results demonstrate that the proposed network achieves competitive detection accuracy on the HRC-WHU dataset while operating with low computational overhead. This results in an effective balance between accuracy and efficiency, offering a practical solution for deploying cloud detection models in resource-limited scenarios.

## 2. Methodology

**Figure 1:**
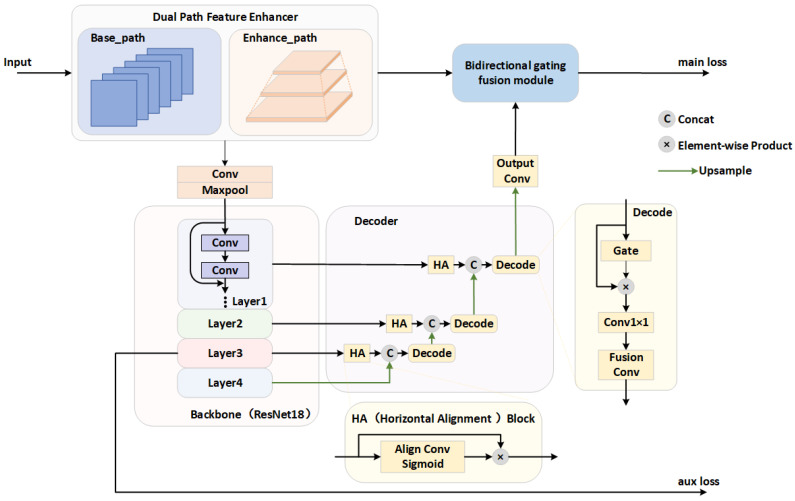
The overall framework of proposed model.

Our proposed lightweight cloud detection network comprises four core components: a Dual-Path Feature Enhancer, a ResNet-18 backbone, a Progressive Feature Pyramid Decoder, and a Bidirectional Gated Fusion Module. First, the Dual-Path Feature Enhancer extracts rich multi-scale contextual features to minimize reliance on a complex backbone. These features are subsequently encoded into deep semantic representations by the ResNet-18 backbone. The Progressive Feature Pyramid Decoder then hierarchically decodes the backbone features, while the Bidirectional Gated Fusion Module adaptively merges the decoder’s deep semantic features with the multi-scale features from the front-end enhancer through gated attention and dynamic convolution. This integrated architecture achieves an effective balance between detection accuracy and computational efficiency. Figure 1 depicts the overall framework of the model.

### 2.1. Overall Design Rationale

The proposed architecture follows a progressive refinement pipeline where each component addresses a specific challenge and its output serves as optimized input for the subsequent module.

We begin with raw remote sensing images that contain complex backgrounds, noise, and varying cloud scales. Directly feeding such inputs into a deep backbone would force it to simultaneously handle low-level denoising and high-level semantic abstraction, competing objectives that reduce efficiency. The dual-path feature enhancer therefore first performs low-level feature refinement, suppressing background clutter while amplifying salient primitives such as cloud edges and thin clouds. This provides a cleaner, more structured representation for the backbone.

With the enhancer handling low-level extraction, the ResNet-18 backbone can focus its limited capacity on building hierarchical semantic representations through progressive downsampling, generating a multi-scale feature pyramid. This functional decoupling, where the enhancer handles spatial primitives and the backbone handles semantic abstraction, justifies the use of a lightweight backbone without sacrificing performance.

The backbone’s feature pyramid contains rich semantics but at varying resolutions. Directly concatenating these features often leads to semantic misalignment between levels. The progressive decoder addresses this through top-down recursive fusion: deeper semantic features are gradually refined with shallower spatial information, while horizontal alignment ensures feature consistency before each fusion step.

Finally, we must integrate two semantically distinct streams: the enhancer’s output rich in spatial primitives and the decoder’s output rich in semantic context. The bidirectional fusion module projects both streams into a unified space, applies gated attention for channel-wise adaptive recalibration, and uses dynamic convolution for sample-adaptive cross-stream interactions, ensuring the fused representation leverages the strengths of both streams.

In essence, the architecture progresses from low-level refinement to hierarchical semantics, then to consistent multi-scale decoding, and finally to adaptive cross-stream fusion, with each component motivated by the specific needs of its position in this pipeline.

### 2.2. Dual-Path Feature Enhancer

We propose a hierarchical dual-path feature enhancement architecture that progressively integrates multi-level features through attention-guided enhancement and gated fusion mechanisms.

The concept of dual-path design has been explored in prior works such as BiSeNet [39], where spatial and context paths operate in parallel to preserve details and capture context. However, our Dual-Path Enhancer differs fundamentally. First, it is positioned as a front-end preprocessing module before the backbone, rather than a parallel branch throughout the network. Second, its purpose is low-level feature refinement, enabling the lightweight backbone to focus solely on semantic modeling. This serialized decoupling contrasts with BiSeNet’s parallel complementarity. Third, its output is later fused with decoder features via bidirectional gating, rather than used directly for prediction. These distinctions position our enhancer as an input refinement mechanism rather than a segmentation backbone.

**Figure 2:**
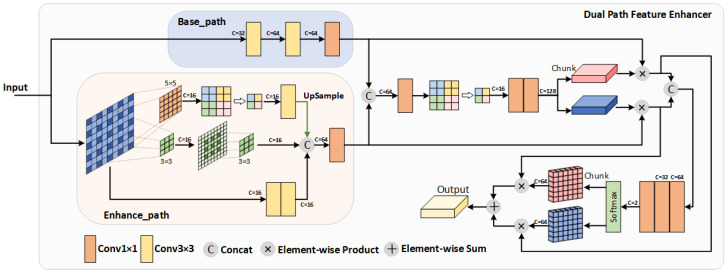
Schematic representation of the Dual-Path Feature Enhancer.

The entire process can be referenced in Figure 2. This design enables adaptive extraction of complementary information from different feature sources: one path preserves global structural information to provide a stable foundation, while the other focuses on extracting and amplifying local detail features (e.g., thin clouds and edge details) that may be overlooked in the basic representation. Separating them early prevents the dilution of global structure by local details, allowing each path to specialize before fusion, after which fine-grained weight adjustment optimizes the final feature representation.

Let the base feature map be $B\in R^{C\times H\times W}$, and the enhanced feature map be $E\in R^{C\times H\times W}$, where *C*, *H*, and *W* represent the number of channels, height, and width, respectively. The complete processing pipeline comprises two core stages:

#### 2.2.1. Attention Feature Enhancement Stage

First, the original features are recalibrated through an attention mechanism. This stage generates spatial-channel adaptive attention weights that enhance useful features while suppressing redundant information:

(1) $$A_{b},A_{e}=\mathrm{Split}\left(A_{\phi }\left(F_{\theta }\left(\mathrm{Concat}\left(B,E\right)\right)\right)\right)$$

Here, $F_{\theta }$ is the feature fusion module implemented as a lightweight 1 × 1 convolutional layer that reduces the concatenated dual-path features (2C channels) to a compact representation of *C* channels, followed by batch normalization and ReLU activation. This design enables efficient cross-path information exchange while maintaining computational efficiency. $A_{\phi }$ denotes the attention module, which first applies global average pooling to aggregate spatial information, then captures channel-wise dependencies through a two-layer bottleneck structure (with reduction ratio 4), and finally generates spatial-channel attention weights via a sigmoid activation. The output is split into $A_{b}$ and $A_{e}$, corresponding to attention weights for the base and enhanced paths respectively. This mechanism allows the model to adaptively emphasize informative channels and spatial regions while suppressing redundant ones.

The enhanced features are then obtained through element-wise multiplication:

(2) $$\begin{array}{c} B_{\mathrm{enhanced}}=B⊙A_{b}\\ E_{\mathrm{enhanced}}=E⊙A_{e} \end{array}$$

#### 2.2.2. The Gating Fusion Stage

While attention provides spatial-channel recalibration, adaptively balancing the relative contributions of the two enhanced streams remains a challenge. To address this, we introduce a gating mechanism that learns optimal fusion weights based on the combined feature context:

(3) $$G_{b},G_{e}=\mathrm{Split}\left(G_{\psi }\left(F_{\theta }\left(\mathrm{Concat}\left(B_{\mathrm{enhanced}},E_{\mathrm{enhanced}}\right)\right)\right)\right)$$

where $G_{\psi }$ denotes the gating weight generation module, which consists of two sequential 1 × 1 convolutional layers: the first reduces the channel dimension from *C* to C/2 with ReLU activation, and the second produces a 2-channel gating map. A Softmax activation is applied along the channel dimension to normalize the gate weights, yielding $G_{b}$ and $G_{e}$ that satisfy $G_{b}+G_{e}=1$. This ensures the two feature streams compete proportionally, with their relative importance adaptively determined per spatial location. This design enables the model to dynamically determine how much each feature stream should contribute based on local context, rather than using fixed fusion ratios.

The final output is then obtained through gated weighted summation, which fuses the two streams according to the learned gate weights:

(4) $$O_{\mathrm{enhanced}}=B_{\mathrm{enhanced}}⊙G_{b}+E_{\mathrm{enhanced}}⊙G_{e}$$

This weighted summation ensures that the final representation preserves both global structural stability from the base path and local detail sensitivity from the enhanced path, with their relative importance adaptively adjusted per spatial location, a key advantage over simple concatenation or addition.

### 2.3. Backbone

In the proposed architecture, the backbone network serves the crucial function of extracting hierarchical semantic features from preprocessed inputs. After thorough evaluation of the trade-off between computational efficiency and representational capacity, ResNet-18 was selected as the foundational backbone. This choice is justified by the fact that the preceding dual-path enhancement module already generates rich and well-structured primary features. By performing initial feature extraction and refinement, it reduces the complexity of the input space and provides the backbone with a cleaner, more informative starting point. Consequently, the backbone is relieved from low-level feature learning responsibilities and can focus its limited capacity on building hierarchical semantic feature pyramids, making a lightweight architecture like ResNet-18 sufficient for the task.

The ResNet-18 backbone comprises four consecutive residual layers that collectively generate a multi-resolution feature pyramid through progressive downsampling. The forward propagation process can be formally described as follows:

(5) $$\begin{array}{c} F_{1}=L_{1}\left(X_{\mathrm{input}}\right)\in R^{64\times \frac{H}{4}\times \frac{W}{4}}\\ F_{2}=L_{2}\left(F_{1}\right)\in R^{128\times \frac{H}{8}\times \frac{W}{8}}\\ F_{3}=L_{3}\left(F_{2}\right)\in R^{256\times \frac{H}{16}\times \frac{W}{16}}\\ F_{4}=L_{4}\left(F_{3}\right)\in R^{512\times \frac{H}{32}\times \frac{W}{32}} \end{array}$$

Here, $L_{i}$ represents the *i*-th residual layer, and the output feature set $F=\{F_{1},F_{2},F_{3},F_{4}\}$ constitutes a feature pyramid covering resolutions from 1/4 to 1/32. This multi-scale feature representation provides rich spatial and semantic information for the subsequent decoder and segmentation head.

To accelerate convergence and enhance generalization capability, the backbone network is initialized with weights pre-trained on the large-scale ImageNet dataset. This initialization strategy leverages generic feature representations learned from diverse visual tasks, effectively mitigating overfitting risks when training data is limited.

### 2.4. Progressive Feature Pyramid Decoder

To effectively leverage the multi-scale feature pyramid produced by the backbone network, this study designs a Progressive Feature Pyramid Decoder. Through lateral connections, progressive fusion, and gating mechanisms, the decoder achieves top-down, multi-level feature integration and ultimately produces high-quality segmentation feature maps.

#### 2.4.1. Horizontal Connection and Feature Alignment

Let $\left\{F_{1},F_{2},F_{3},F_{4}\right\}$ represent the feature pyramid output by the backbone network, where $F_{i}\in R^{C_{i}\times H_{i}\times W_{i}}$. First, features at each level are projected into a unified feature space through lateral convolutional layers:

(6) $$L_{i}=\mathrm{ReLU}\left(\mathrm{BN}\left({\mathrm{Conv}}_{1\times 1}\left(F_{i}\right)\right)\right)\in R^{C_{d}\times H_{i}\times W_{i}}$$

where $C_{d}$ denotes the decoder channel dimension, and $L_{i}$ represents the lateral features at the *i*-th level. This operation standardizes encoder features with varying channel numbers into a consistent dimensionality, thereby facilitating subsequent fusion operations.

#### 2.4.2. Top-Down Progressive Fusion

The decoding process begins with the deepest semantic features and progressively incorporates shallow spatial information. Let $R_{i}$ denote the refined features at the *i*-th level.

A key challenge in multi-scale fusion is the semantic gap between features from different levels, where deeper features carry rich semantics but coarse spatial details, while shallower features contain fine spatial information but limited semantics. To address this, we first align both resolution and semantic distribution before fusion.

For i=3,2,1 (top-down):

(7) $$U_{i+1}=\mathrm{Interpolate}\left(R_{i+1},\mathrm{size}=\left(H_{i},W_{i}\right)\right)$$

This upsampling step resolves the resolution mismatch by aligning deeper features to the spatial size of the current level.

(8) $$A_{i}=H_{i}\left(L_{i}\right)$$

Here $H_{i}$ further aligns the semantic distribution of lateral features to match that of the upsampled deeper features, reducing semantic discrepancies that would otherwise degrade fusion quality. This two-step alignment ensures that subsequent fusion operates on features that are both spatially and semantically consistent.

(9) $$C_{i}=\mathrm{Concat}\left[U_{i+1},A_{i}\right]\in R^{2C_{d}\times H_{i}\times W_{i}}$$

(10) $$G_{i}=G_{i}\left(C_{i}\right)$$

where $G_{i}$ represents the gated skipping connection module that adaptively regulates feature contributions from different sources.

(11) $$R_{i}=P_{i}\left(G_{i}\right)$$

where $P_{i}$ is the progressive fusion block that further optimizes feature representations.

The initial condition is set as $R_{4}=L_{4}$, meaning the deepest lateral features serve as the starting point for fusion.

#### 2.4.3. Output Feature Generation

Following the top-down progressive integration, the highest-resolution refined feature $R_{1}$ is obtained. The decoder output is subsequently generated through an output projection layer:

(12) $$D_{\mathrm{output}}=W_{\mathrm{output}}\left(R_{1}\right)$$

where $W_{\mathrm{output}}$ represents a two-stage convolutional projection that progressively compresses the feature dimension from $C_{d}$ to $C_{d}/4$.

This decoder design offers several notable advantages: First, the lateral connections effectively preserve spatial details from the encoder. Second, the progressive fusion mechanism ensures smooth propagation of semantic information across different levels. Third, the gated skip connections enhance the model’s capacity for adaptive feature importance selection. Finally, the horizontal alignment module improves semantic consistency among features. From the perspective of feature optimization and gradient propagation, these design choices collectively ensure feature consistency by aligning multi-scale representations before fusion, and facilitate gradient flow through recursive connections that create shorter paths for backpropagation. These are key advantages over simpler decoder architectures.

### 2.5. Bidirectional Gated Fusion Module

**Figure 3:**
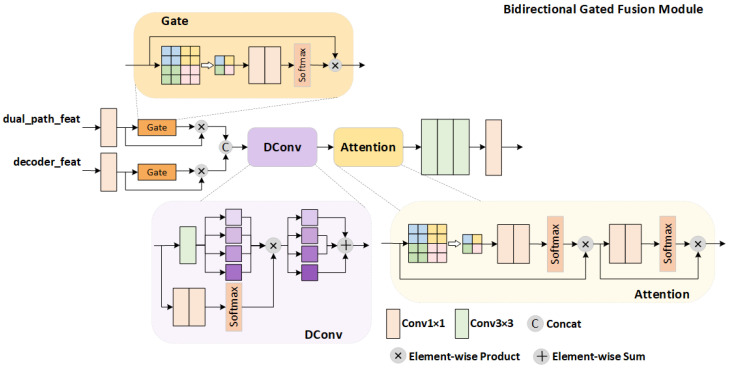
Schematic representation of the Bidirectional Gated Fusion Module.

To effectively integrate the multi-scale features extracted by the front-end enhancer with the deep semantic features from the backbone decoder, this study proposes a Bidirectional Gated Fusion Module, as shown in Figure 3. This module achieves multi-level adaptive feature fusion through bidirectional feature projection, gated attention mechanisms, and dynamic convolution operations, significantly enhancing the richness and discriminability of feature representations. This design aligns with the broader trend in the detection literature that leverages adaptive feature aggregation to enhance representation quality [40].

#### 2.5.1. Feature Projection and Spatial Alignment

Given dual-path features $F_{\mathrm{dual}}\in R^{C_{d}\times H_{d}\times W_{d}}$ and decoder features $F_{\mathrm{dec}}\in R^{C_{c}\times H_{c}\times W_{c}}$, the features are first projected into a unified fusion space through 1 × 1 convolutional layers:

(13) $$\begin{array}{cc} P_{\mathrm{dual}} & ={\mathrm{Conv}}_{1\times 1}\left(F_{\mathrm{dual}}\right)\in R^{C_{f}\times H\times W}\\ P_{\mathrm{dec}} & ={\mathrm{Conv}}_{1\times 1}\left(F_{\mathrm{dec}}\right)\in R^{C_{f}\times H\times W} \end{array}$$

where $C_{f}$ denotes the predefined fusion channel dimension. When spatial dimensions between input features are mismatched, bilinear interpolation is applied for spatial alignment:

(14) $$P_{\mathrm{dual}}^{\prime }=\mathrm{Interpolate}\left(P_{\mathrm{dual}},\mathrm{size}=\left(H_{c},W_{c}\right)\right)$$

This alignment operation ensures spatial consistency for subsequent fusion steps and establishes the foundation for effective feature interaction.

#### 2.5.2. Bidirectional Gated Attention Mechanism

The core innovation of this module resides in its bidirectional gated attention mechanism, which generates channel-adaptive gating weights by leveraging global contextual information. Specifically, channel-wise statistics are first extracted through global average pooling:

(15) $$z_{\mathrm{dual}}=\mathrm{GAP}\left(P_{\mathrm{dual}}^{\prime }\right)=\frac{1}{H\times W}\sum_{i=1}^{H}\sum_{j=1}^{W}P_{\mathrm{dual}}^{\prime }\left(i,j\right)$$

To model the relationships between channels and generate adaptive weights, we pass the channel descriptors through a bottleneck structure that captures inter-channel dependencies:

(16) $$W_{\mathrm{dual}}=\sigma \left(W_{2}\cdot \mathrm{ReLU}\left(W_{1}\cdot z_{\mathrm{dual}}\right)\right)$$

The reduction ratio of 8 in the bottleneck compresses channel information to learn compact representations of channel relationships, while the sigmoid activation produces soft gating values in (0, 1). This design enables the model to emphasize interdependent channels while suppressing less relevant ones based on global context. Where $W_{1}\in R^{\left(C_{f}/8\right)\times C_{f}}$ and $W_{2}\in R^{C_{f}\times \left(C_{f}/8\right)}$ are learnable parameters, and σ denotes the sigmoid activation function. The same computational process is applied to the decoder features to generate $W_{\mathrm{dec}}$.

Ultimately, the gating weights are applied to their corresponding features through element-wise multiplication:

(17) $$\begin{array}{cc} G_{\mathrm{dual}} & =P_{\mathrm{dual}}^{\prime }⊙W_{\mathrm{dual}}\\ G_{\mathrm{dec}} & =P_{\mathrm{dec}}⊙W_{\mathrm{dec}} \end{array}$$

where ⊙ denotes the element-wise multiplication operation.

This mechanism enables the model to adaptively emphasize information-rich channels while suppressing redundant or noisy ones, thereby achieving fine-grained regulation at the feature level.

#### 2.5.3. Dynamic Feature Fusion

To further enhance the fusion performance, this module introduces a dynamic convolution operation. First, the bidirectional gated features are concatenated along the channel dimension. While gated attention provides channel-wise selection, effectively fusing the two streams also requires capturing spatial interactions that vary across samples. To address this, we introduce dynamic convolution that adapts its parameters to each input:

(18) $$F_{\mathrm{enhanced}}=A_{e}\left(D\left(\mathrm{Concat}\left[G_{\mathrm{dual}},G_{\mathrm{dec}}\right]\right)\right)\in R^{2C_{f}\times H\times W}$$

Unlike standard convolution with fixed kernels, D generates kernel weights conditioned on the input features, enabling sample-adaptive cross-stream interactions. This allows the model to capture varying feature relationships across different images (e.g., different cloud types or backgrounds), followed by an enhanced attention module $A_{e}$ for further refinement.

#### 2.5.4. Output Transformation and Feature Refinement

Finally, high-quality fused features are generated through the output projection layer:

(19) $$O={\mathrm{Dropout}}_{0.1}\left(\mathrm{ReLU}\left(\mathrm{BN}\left({\mathrm{Conv}}_{3\times 3}\left(F_{\mathrm{enhanced}}\right)\right)\right)\right)$$

The module returns identical feature pairs (O,O) for subsequent processing, maintaining feature consistency while providing sufficient semantic information.

The bidirectional gated fusion module offers several significant advantages: First, the bidirectional projection mechanism ensures effective alignment and interaction of features from different sources. Second, the global context-based gated attention enables adaptive feature selection at the channel level. Third, the dynamic convolution operation enhances the model’s adaptability to input variations. Finally, the fully differentiable end-to-end design supports efficient gradient propagation and parameter optimization. Experimental results demonstrate that this module effectively improves feature representation quality and discriminability in complex visual understanding tasks.

### 2.6. Loss Function

This study employs a joint primary-auxiliary loss optimization strategy to balance the learning of deep semantic features and shallow detail features. The overall loss function comprises the main segmentation loss and an auxiliary supervision loss:

(20) $$L_{\mathrm{total}}=L_{\mathrm{main}}+\alpha \cdot L_{\mathrm{aux}}$$

where both $L_{\mathrm{main}}$ and $L_{\mathrm{aux}}$ employ the cross-entropy loss function. The auxiliary loss weight α follows a linear decay schedule and is dynamically adjusted throughout the training process:

(21) $$\alpha \left(t\right)=\alpha_{\mathrm{begin}}+\frac{\alpha_{\mathrm{end}}-\alpha_{\mathrm{begin}}}{T_{\mathrm{total}}}\cdot t$$

Here, *t* denotes the current training epoch, $T_{\mathrm{total}}$ represents the total number of training epochs, while $\alpha_{\mathrm{begin}}$ and $\alpha_{\mathrm{end}}$ indicate the initial and final weight values, respectively. Based on empirical tuning, we set $\alpha_{\mathrm{begin}}=0.5$ and $\alpha_{\mathrm{end}}=0.4$, as this linear decay schedule showed better convergence than fixed weights in preliminary experiments. This design allows the model to initially rely more heavily on auxiliary supervision for training stability, while progressively shifting focus toward primary task optimization in later stages.

## 3. Experimental Setup and Results

This section elaborates on the datasets employed, parameter configurations set, and accuracy evaluation criteria adopted in the experiments. Following this, we conducted ablation experiments aimed at verifying the effectiveness and contribution of the modules. Lastly, we performed an in-depth qualitative and quantitative comparative analysis between the proposed model and other state-of-the-art methods across multiple datasets, to comprehensively evaluate its performance and robustness.

### 3.1. Dataset

**Figure 4:**
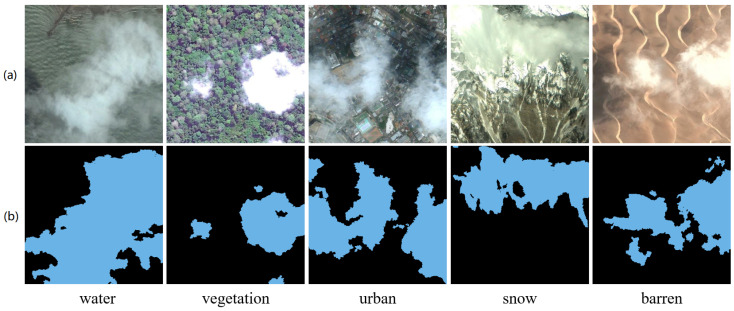
Cloud images with different land object types in HRC_WHU dataset. (**a**) Image, (**b**) Ground Truth.

The HRC_WHU dataset [41], created by the SENDIMAGE Lab of Wuhan University, is designed for cloud detection tasks in high-resolution remote sensing images. The dataset comprises 150 high-resolution images sourced from Google Earth, covering various regions of the globe and featuring five primary land object types: water, vegetation, urban, snow and barren, as presented in Figure 4. In our experiments, the HRC_WHU dataset was cropped into mutually non-overlapping image segments, each measuring 256×256 pixels. The training set consists of 8800 patches, while the test set comprises 2800 patches.

In the HR_WHU dataset ground truth masks, pixel values 0 and 1 represent background and cloud, respectively. Thus, in Figure 4, black pixels in row b correspond to background.

In order to enhance the diversity of the dataset and promote the generalization ability of the model, during the training phase, we employ random cropping and scaling techniques to resize the images to the specified size. The area ratio of the cropped area is randomly varied between 75% and 15%.

### 3.2. Experimental Setup and Evaluation Metrics

Our experiments were conducted on a workstation with an NVIDIA RTX 3090 GPU (24 GB VRAM), using PyTorch 1.12.1 with CUDA 11.6 and cuDNN 8.4. All models were trained for 100 epochs in FP32 precision, with a batch size of 16 constrained by GPU memory. We adopted the AdamW optimizer (initial learning rate: $2\times 10^{-4}$, $\beta_{1}$ = 0.9, $\beta_{2}$ = 0.999) and applied L2 weight decay (λ = 0.01).

A segmented learning rate scheduling mechanism is employed, comprising a warm-up phase followed by a cosine annealing phase:

Warm-up phase (when $t<T_{\mathrm{warm}}$):

(22) $$\eta \left(t\right)=\eta_{\mathrm{init}}\cdot \frac{t}{T_{\mathrm{warm}}}$$

Cosine annealing phase (when $t\geq T_{\mathrm{warm}}$):

(23) $$\begin{array}{cc} \eta (t) & =\eta_{\min}+\left(\eta_{\max}-\eta_{\min}\right)\cdot \delta \left(t\right)\\ \delta (t) & =0.5+0.5\cdot \cos\left(\pi \cdot \frac{t-T_{\mathrm{warm}}}{T_{\mathrm{total}}-T_{\mathrm{warm}}}\right) \end{array}$$

Here, $T_{\mathrm{warm}}$ represents the number of warm-up epochs, $\eta_{\mathrm{init}}$ denotes the initial learning rate, while $\eta_{\min}$ and $\eta_{\max}$ specify the minimum and maximum bounds of the learning rate, respectively. This design ensures training stability through the warm-up phase while achieving fine-tuned optimization via cosine annealing in later stages.

To ensure reproducibility, we fixed random seeds and reported mean results over 3 runs.

The performance of cloud detection results is evaluated comprehensively using a variety of widely adopted quantitative assessment metrics, namely accuracy, precision, recall, F1-score, and intersection over union (IoU). Below are the definitions of these key metrics:

(24) $$\mathrm{accuracy}=\frac{\mathrm{TP}+\mathrm{TN}}{\mathrm{TP}+\mathrm{TN}+\mathrm{FP}+\mathrm{FN}}$$

(25) $$\mathrm{precision}=\frac{\mathrm{TP}}{\mathrm{TP}+\mathrm{FP}}$$

(26) $$\mathrm{recall}=\frac{\mathrm{TP}}{\mathrm{TP}+\mathrm{FN}}$$

(27) $$F1\text{-}\mathrm{score}=\frac{2\times \mathrm{precision}\times \mathrm{recall}}{\mathrm{precision}+\mathrm{recall}}$$

(28) $$\mathrm{IoU}=\frac{\mathrm{TP}}{\mathrm{TP}+\mathrm{FP}+\mathrm{FN}}$$

where TP, TN, FP and FN stand for true positive, true negative, false positive and false negative, respectively. MIoU is equal to the arithmetic mean of IoU of all types (cloud and background).

### 3.3. Analysis of Feature Representation in Dual Paths

Experimental results from the dual-path network reveal a clear functional divergence, which is analyzed as follows.

#### 3.3.1. Mechanism of Feature Representation and Attention

**Figure 5:**
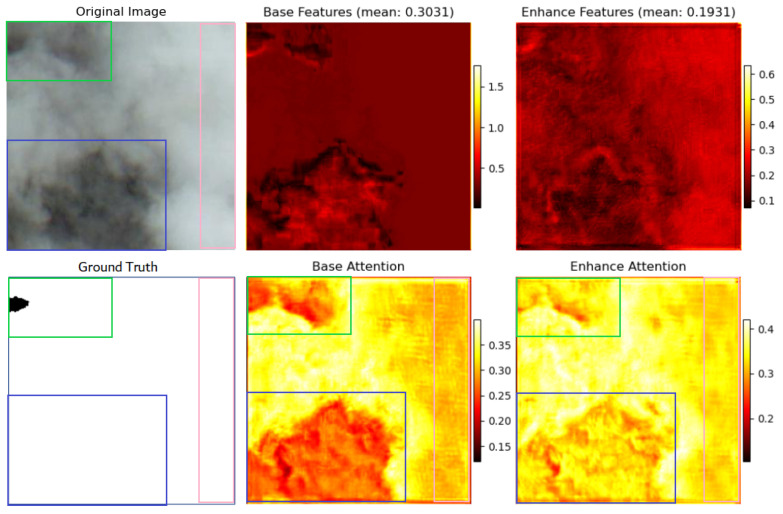
Comparison of feature maps and attention visualization of the dual-path network.

As presented in Figure 5, the Base branch’s feature map exhibits a relatively high mean value (0.3031), indicating generally large activated pixel values and strong responses. The visualization results reveal that this branch retains the majority of the original image’s structural and content information with clear contours, suggesting its role in learning global and fundamental representations. In contrast, the Enhance branch’s feature map has a lower mean value (0.1931), implying sparser feature activation and a more uniform overall response compared to the Base branch. However, it shows relatively higher activation in regions with significant texture variations. This phenomenon indicates that the Enhance branch is functionally geared not toward global reconstruction, but toward extracting and amplifying local detail information within the image.

Furthermore, the attention map analysis corroborates this functional divergence. While both branches attend to cloud regions as expected for a cloud detection task, a closer examination of their activation patterns reveals distinct yet complementary specializations.

The attention weights of the base branch are more concentrated in areas with rich information and distinct texture features, such as the cloud center regions. In contrast, the enhanced branch exhibits a more dispersed attention pattern throughout the image, showing a more significant response in low-contrast and texture-weakened areas (such as thin clouds), features that are crucial for accurately classifying cloud layers. This is clearly demonstrated in the green and blue boxes in Figure 5, where the thin cloud regions in the original image show significantly higher attention values in the enhanced branch, confirming its role in restoring fine texture details. Due to the smoothing process in the base feature extraction, the activation level of the base branch is lower in such areas. In the thick and uniform cloud regions marked by the pink boxes (with a smooth appearance and minimal texture features), the attention value of the enhanced branch is slightly higher than that of the base branch, indicating that it can meaningfully contribute to feature representation even in texture-less areas. Overall, the enhanced branch consistently maintains higher attention across all cloud types, especially in thin cloud regions where detail restoration is most critical, and also plays an important role in smooth and texture-less areas. Although the base branch shows slightly lower absolute attention values, it provides the essential structural background. When combined with the continuous enhancement effect of the enhanced branch, it enables more comprehensive cloud detection than either branch acting alone.

#### 3.3.2. Attention and Entropy

**Figure 6:**
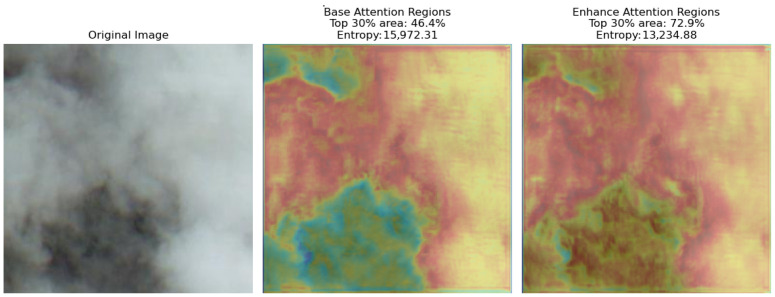
Comparison of attention region of the dual-path network.

As evidenced by the data in Figure 6, the Base and Enhance branches exhibit distinct attention patterns and information entropy profiles, underscoring their complementary roles. The Base branch, characterized by a dispersed attention distribution (with the top 30% high-attention areas covering only 46.4% of the image) and high information entropy (15,972.31), ensures comprehensive coverage and stable preservation of the global structure. In contrast, the Enhance branch employs a highly concentrated attention mechanism (the top 30% high-attention areas cover 72.9%), coupled with a lower information entropy (13,234.88), which facilitates the precise localization and enhanced processing of critical regions. This collaborative dynamic between the two branches provides an effective solution for image processing tasks, successfully balancing global fidelity with local refinement.

#### 3.3.3. Correlation Analysis

**Figure 7:**
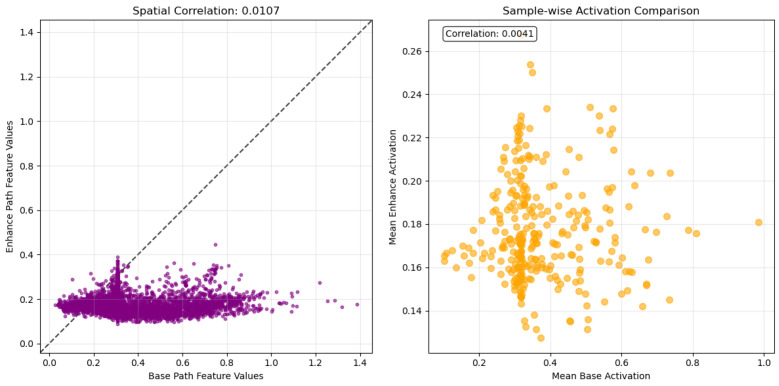
Correlation analysis of the dual-path network.

As evidenced by the data in Figure 7, the analysis of spatial correlation and activation values reveals a high degree of functional differentiation and complementarity between the Base and Enhance branches, as evidenced by their extremely low spatial correlation (0.0107) and activation comparison value (0.0041). These low values reflect structured complementarity rather than noise, as the scatter plot shows clustered distributions (indicating systematic spatial specialization) and the sample-wise comparison reveals consistent activation patterns across images. This statistical evidence confirms their distinct yet complementary roles: the Base branch serves as a foundation, providing a global and stable structural representation, whereas the Enhance branch acts as a specialized enhancer. It is responsible for precisely restoring and enhancing local details and weak features that are overlooked or smoothed out by the Base branch through selective and subtle activation modulation. The combined strategy of a “global foundation” with “local modulation” thus establishes an effective paradigm for high-quality image processing.

### 3.4. Decoding the Role of the Bidirectional Gated Fusion Module

**Figure 8:**
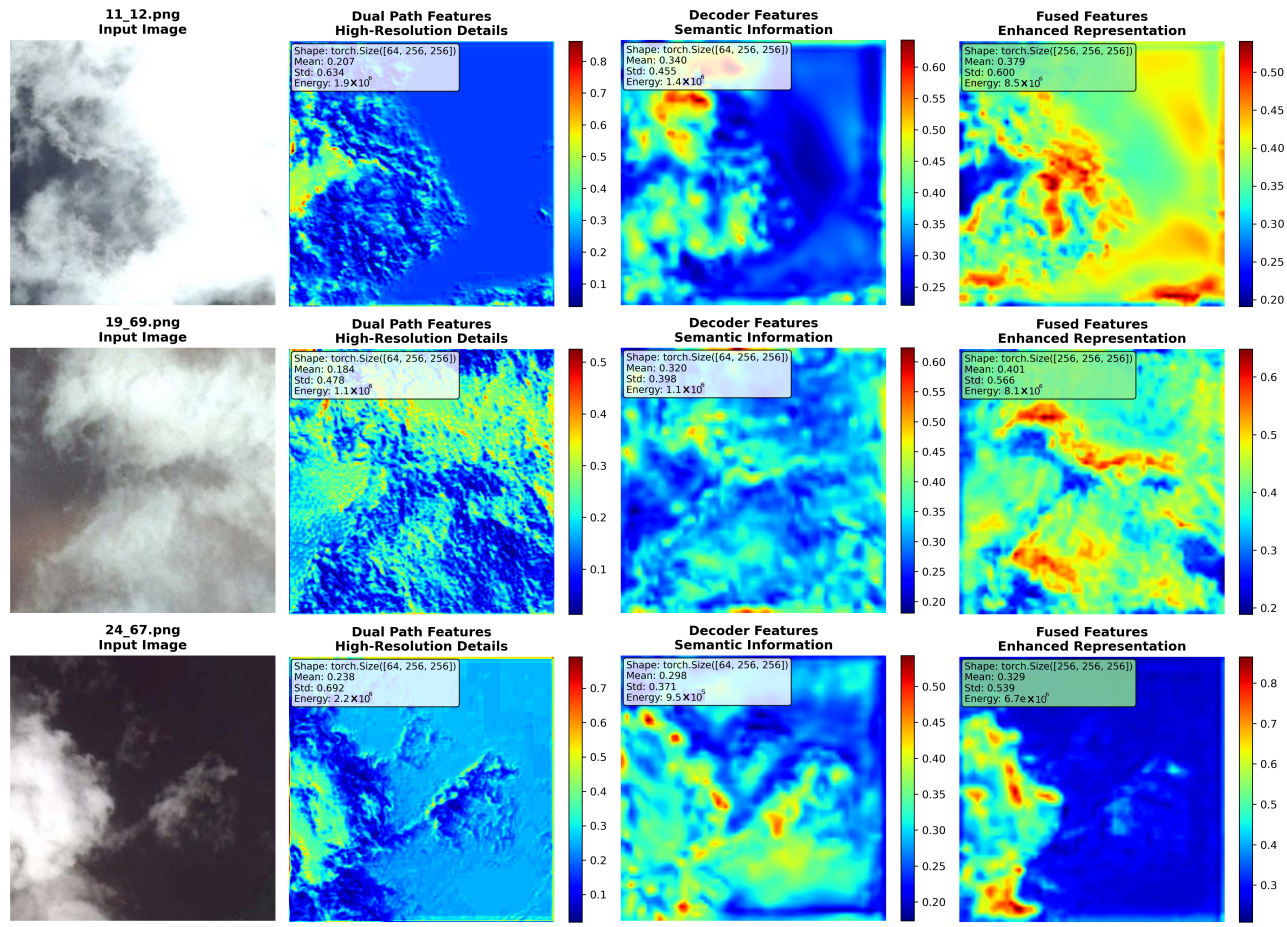
Visualized feature selection and integration in the Bidirectional Gated Fusion Module.

The proposed bidirectional gated fusion module demonstrates exceptional feature fusion capability across multiple test samples, As presented in Figure 8. Its hierarchical design first applies a bidirectional gated attention mechanism to weight high-resolution details and semantic information selectively. This “feature purification” is crucial for the subsequent deep fusion.

Quantitative results on three representative samples (11_12.png, 19_69.png, 24_67.png) confirm the module’s efficacy. It achieves significant feature energy enhancement (by factors of 6.26×, 7.37×, and 7.04×, respectively), a nonlinear effect primarily attributable to the dynamic convolution layer. This layer intelligently integrates gated features through learnable kernel parameters, producing a synergistic effect beyond simple superposition.

Furthermore, the module optimizes the feature distribution via an enhanced attention mechanism, improving semantic consistency while preserving rich details. The stability of feature diversity is significantly increased (by factors of 1.32×, 1.42×, and 1.45×). This is reflected in the standard deviation of the features, which is optimized from 0.455 in the decoder features to a range of 0.5386–0.6002 after fusion, striking an ideal balance between distribution breadth and stability. Notably, sample 24_67.png achieved a 7.04× energy gain and a 1.45× diversity improvement despite a relatively low initial feature mean (0.3291), underscoring the module’s robust adaptability.

Finally, an output projection layer with 256-channel capacity ensures the enhanced features possess sufficient expressive power for subsequent cloud detection tasks, providing high activation intensity, rich diversity, and strong discriminability.

In summary, the module constructs an efficient pipeline through a serial process of “gated selection → dynamic fusion → attention refinement → capacity retention.” Its core advantage lies in performing adaptive, nonlinear, multi-dimensional deep fusion, thereby combining the “breadth” of high-resolution details with the “depth” of semantic information to generate high-quality feature representations with significant gains in both energy and diversity.

### 3.5. Ablation Experiments

To validate the individual contributions of the proposed dual-path architecture, gated fusion module, and auxiliary output mechanism, we conducted systematic ablation studies. As shown in Table 1, all experiments used identical datasets and training settings. We quantitatively evaluated performance improvements by incrementally adding each module.

Our baseline (M1) is a standard U-Net architecture with a ResNet-18 encoder and a basic decoder consisting of upsampling layers and convolutional blocks. We first introduced the dual-path feature extraction module (M2). This addition yielded a significant performance gain, increasing the mIoU from 89.04% to 90.91% (+1.87 pp). This confirms the effectiveness of the dual-path design, which captures both high-level semantics and multi-scale details through its parallel base and enhance paths, thereby constructing a more discriminative feature representation.

Building upon the dual-path architecture, we integrated the bidirectional gated fusion module (M3). The results show that the mIoU was further improved to 92.02% (+1.11 pp over M2). This indicates that simple concatenation or addition is suboptimal for fusion. In contrast, the gating mechanism adaptively learns the interdependencies between the two paths and dynamically adjusts their contribution weights, leading to more efficient and robust feature fusion that fully leverages their complementary advantages.

Finally, we incorporated an auxiliary output supervision into M3 to form our complete proposed model (M4). This model achieved optimal performance across all metrics, with mIoU, F1-score, and OA reaching 92.82%, 96.27%, and 96.31%, respectively. Although the absolute improvement from the auxiliary output was smaller, it played a critical role in stabilizing the training process and mitigating gradient vanishing in deep networks, thereby contributing to the final performance.

In summary, our ablation study demonstrates the cumulative benefit of each component. The dual-path architecture was the primary source of improvement (contributing approximately 49.5% of the total mIoU gain), establishing the model’s fundamental capability. The gated fusion module was a key optimization (contributing approximately 29.4%), fully unlocking the potential of the dual paths.The auxiliary output provided essential training stability and regularization (contributing approximately 21.1%). These results collectively validate the necessity and collaborative efficacy of every proposed module in our framework.

### 3.6. Comparison Study

To comprehensively evaluate our model, we conducted a comparative analysis on the HRC_WHU dataset against several state-of-the-art methods. The selected competitors, which include CNN-based (AFMUnet [12], BABFNet [18]), Transformer-based (CSDFormer [28]), and hybrid architectures (TransGA [36], SwinCloud [37], DFC-Net [38]), encompass the main network paradigms currently prevailing in the field. This comprehensive comparison validates the synergistic effect of our two core innovations.First, the dual-path feature enhancer enriches input representations at the front-end, allowing for a lightweight backbone. Second, the Bidirectional Gated Fusion Module further refines these features, ultimately enhancing fusion accuracy and the model’s discriminative power beyond existing specialized or hybrid designs.

**Figure 9:**
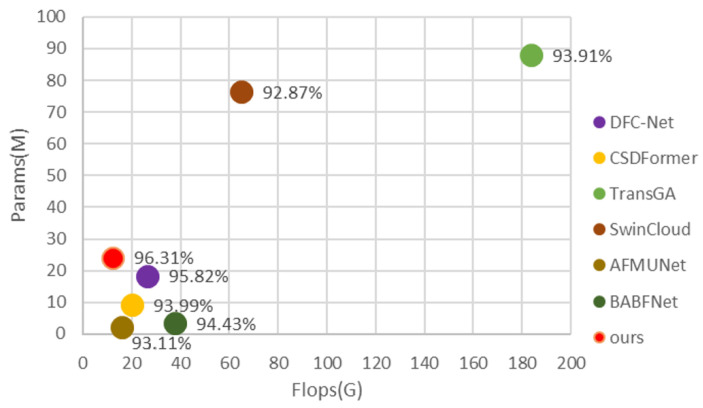
Comparison of model efficiency based on params, OA, and FLOPs.

The proposed model achieves a breakthrough in balancing computational efficiency and classification accuracy. As shown in Figure 9, with a complexity of only 12.04 GFLOPs, our model attains the lowest level among all competitors—approximately 30% lower than the second-most efficient model, AFMUNet (15.74 GFLOPs). Crucially, this exceptional efficiency is achieved without sacrificing accuracy. The model reaches an overall accuracy (OA) of 96.31%, significantly outperforming all compared methods. It surpasses the second-ranked DFC-Net (95.82% OA) by 0.31 percentage points while reducing computational cost by approximately 58%. Furthermore, compared to AFMUNet (93.11% OA), which has similar complexity, our model delivers a substantial accuracy gain of 3.02 percentage points. This demonstrates that our innovative architecture enables highly efficient parameter utilization, delivering optimal classification performance with minimal computational burden, thus presenting an ideal solution for high-precision vision tasks in resource-constrained environments.

As comprehensively summarized in Table 2, our model achieves state-of-the-art performance across all evaluation metrics. It attains an mIoU of 92.82%, an F1-score of 96.27%, a precision of 96.26%, a recall of 96.29%, and an overall accuracy (OA) of 96.31%. Notably, it surpasses the strong DFC-Net baseline by a significant margin of 1.83 percentage points in the critical mIoU metric. Furthermore, our model secures the highest recall while maintaining top-tier precision, demonstrating an effective balance between minimizing false alarms and reducing missed detections. The results show that our method outperforms other advanced models, which represent different architectural paradigms, thereby validating the superiority and effectiveness of the proposed approach.

**Figure 10:**
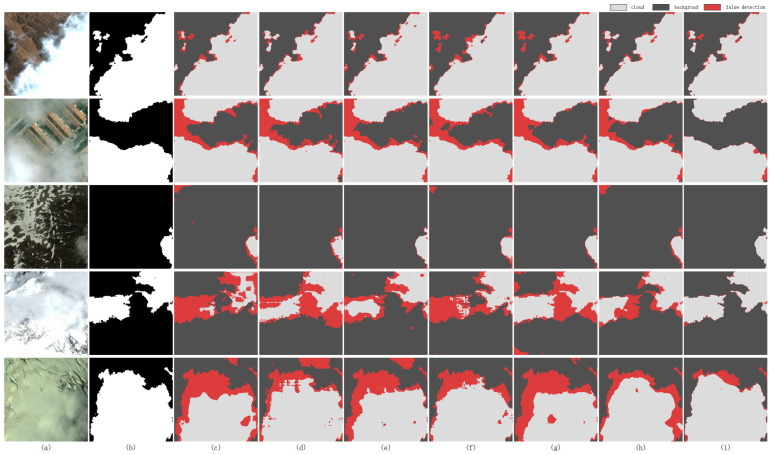
Comparison of cloud detection results using various methods on the HRC_WHU dataset. (**a**) image, (**b**) Ground Truth, (**c**) BABFNet, (**d**) CSDFormer, (**e**) TransGA, (**f**) AFMUnet, (**g**) SwinCloud, (**h**) DFC-Net, (**i**) Ours.

The qualitative comparison in Figure 10 visually underscores the superior performance of our method across various challenging scenarios. In the first row, which presents a clear cloud image, all baseline models demonstrate competent detection performance at a coarse level. However, our proposed method yields significantly finer boundaries and preserves more complete edge details of the cloud bodies. In the second row, depicting an urban scene with thin clouds, our method achieves higher accuracy in capturing both the cloud boundaries and the subtle, semi-transparent regions where ground information is faintly visible, demonstrating a enhanced capability for weak feature extraction.

The coexistence of clouds and snow poses a persistent challenge in cloud detection. As shown in the third row, when clouds and snow are spatially separate, existing models retain a basic detection capability. However, when thin clouds overlap with underlying thick snow cover (rows four and five), these models struggle to differentiate between the two, leading to significant missed detections or misclassifications. In stark contrast, our method maintains robust and stable detection performance even in these complex scenarios, substantially outperforming all existing alternatives. While our dual-path design does not explicitly model the distinction between clouds and snow, we attribute this performance gain to the enhanced feature representations from complementary paths (global structure and local details) and the adaptive gating mechanism that dynamically selects the most relevant features for each region. These properties collectively improve discrimination in complex scenes, even without task-specific supervision for cloud-snow separation. Further investigation into explicit cloud-snow discrimination is left for future work.

## 4. Discussion

Advantage. Based on comprehensive experiments and analysis, our model demonstrates three key strengths. First, it achieves a breakthrough in balancing computational efficiency and detection accuracy. Operating at only 12.04 GFLOPs, it attains 96.31% overall accuracy and 92.82% mean IoU on the HRC_WHU dataset, substantially outperforming comparable models and offering a practical solution for compute-constrained onboard or edge deployment.

Second, the model enables efficient multi-level feature extraction and fusion through its dual-path feature enhancer and bidirectional gated fusion module. The front-end enhancer uses a parallel architecture to diversify input representations and reduce dependency on heavy backbones, while the fusion module employs gated attention and dynamic convolution to adaptively combine semantic and spatial features, significantly improving weak feature capture and boundary preservation.

Finally, the architecture exhibits strong robustness in complex scenarios. It consistently maintains high detection accuracy—even with thin clouds, urban landscapes, and cloud-snow coexistence—effectively balancing false alarms and missed detections. Its superior performance across diverse settings validates the effectiveness and general applicability of the proposed method under challenging real-world conditions.

Disadvantage. The proposed model was developed and evaluated exclusively on three-channel RGB imagery, which limits its ability to leverage the rich spectral information available in near-infrared and short-wave infrared bands. In the context of our current RGB-only experiments, this constitutes both a data limitation (the input lacks spectral information) and a model limitation (our architecture is designed for three-channel inputs). This inherently restricts its potential for more refined cloud analysis tasks, such as detailed cloud phase classification (e.g., discriminating between ice and water clouds) or fine-grained cloud type recognition (e.g., distinguishing convective clouds from cumulonimbus). Consequently, the model’s discriminative capability in certain complex scenarios, particularly those where spectral characteristics are critical for accurate detection, remains limited compared to what could be achieved with multispectral or hyperspectral input. Addressing this limitation requires architectural evolution to accommodate multispectral inputs, which we outline as a primary direction in our future work.

## 5. Conclusions and Future Work

In this paper, we propose a novel lightweight network for cloud detection in high-resolution remote sensing imagery, effectively balancing accuracy and efficiency. To address the limitations of existing models that rely on computationally intensive backbones, we introduce two key innovations: a dual-path feature enhancer that enriches multi-scale input representations while reducing backbone dependency, and a bidirectional gated fusion module that adaptively integrates semantic and spatial features during decoding.

Experiments on the HRC_WHU dataset demonstrate that our model achieves state-of-the-art performance, with 96.31% OA and 92.82% mIoU, while requiring only 12.04 GFLOPs—significantly more efficient than comparable methods. The model maintains high precision on challenging features like thin clouds and fine boundaries without sacrificing efficiency.

These results confirm that our approach successfully bridges the gap between detection performance and operational practicality. The proposed network offers an efficient, robust solution suitable for resource-constrained environments such as onboard satellite processing and edge computing, advancing real-time cloud detection capabilities in operational scenarios.

Building on the presented RGB-based framework, future research will explore its extension into multispectral and multi-modal domains. A primary direction is to generalize the architecture for multispectral inputs, specifically utilizing VNIR and SWIR bands to advance edge analysis and precise cloud typing. Concurrently, we plan to evolve the Dual-Path Feature Enhancer into an adaptive multi-modal matching module. This innovation will empower it to extract and fuse complementary features from diverse data sources in a unified high-dimensional space, achieving powerful multi-modal fusion without resorting to modality-specific backbone alterations, thus upholding the model’s lightweight and versatile design principle. 

## Figures and Tables

**Table 1:** Ablation Study Analysis of Different Modules’ Impact on Performance for the HRC_WHU Datasets.

Model	BL	DP	BG	$L_{\mathrm{aux}}$	OA	MIoU	F1
					(%)	(%)	(%)
M1	✓				94.26	89.04	94.21
M2	✓	✓			95.29	90.91	95.23
M3	✓	✓	✓		95.89	92.02	95.84
M4	✓	✓	✓	✓	96.31	92.82	96.27

Note: BL: Baseline. DP: Dual-Path Feature Enhancer. BG: Bidirectional Gated Fusion Module. $L_{\mathrm{aux}}$: Auxiliary Loss.

**Table 2:** Comparison of evaluation metrics of different models on the HRC_WHU dataset.

Model	OA	MIoU	Recall	Precision	F1
	(%)	(%)	(%)	(%)	(%)
BABFNet	94.43	87.92	93.08	94.05	93.56
AFMUnet	93.11	84.47	91.05	92.11	91.58
CSDFormer	93.99	86.72	92.59	93.13	92.86
TransGA	93.91	87.06	92.51	93.63	93.07
SwinCloud	92.87	84.37	90.34	92.69	91.50
DFC-Net	95.82	90.99	95.38	95.18	95.28
ours	96.31	92.82	96.29	96.26	96.27

**Red bold**: Best performance (top-1) in each column. **Blue bold**: Second-best performance (top-2) in each column.

## Data Availability

The raw data supporting the conclusions of this article will be made available by the authors on request.

## References

[1] Shen H., Li X., Cheng Q., Zeng C., Yang G., Li H., Zhang L. (2015). Missing information reconstruction of remote sensing data: A technical review. IEEE Geosci. Remote Sens..

[2] Johnston T., Young S.R., Hughes D., Patton R.M., White D. Optimizing convolutional neural networks for cloud detection. Proceedings of the Machine Learning on HPC Environments.

[3] Liu Y., Wang W., Li Q., Min M., Yao Z. (2022). DCNet: A Deformable Convolutional Cloud Detection Network for Remote Sensing Imagery. IEEE Geosci. Remote Sens. Lett..

[4] Yanan G., Xiaoqun C., Bainian L., Kecheng P. (2020). Cloud detection for satellite imagery using deep learning. J. Phys. Conf. Ser..

[5] Guo Y., Cao X., Liu B., Gao M. (2020). Cloud Detection for Satellite Imagery Using Attention-Based U-Net Convolutional Neural Network. Symmetry.

[6] Ronneberger O., Fischer P., Brox T. (2015). U-net: Convolutional networks for biomedical image segmentation. Medical Image Computing and Computer-Assisted Intervention–MICCAI 2015: 18th International Conference, Munich, Germany, 5–9 October 2015.

[7] Hu K., Zhang D., Xia M. (2021). CDUNet: Cloud Detection UNet for Remote Sensing Imagery. Remote Sens..

[8] Guo J., Yang J., Yue H., Tan H., Hou C., Li K. (2021). CDnetV2: CNN-Based Cloud Detection for Remote Sensing Imagery with Cloud-Snow Coexistence. IEEE Trans. Geosci. Remote Sens..

[9] Zhang J., Wu J., Wang H., Wang Y., Li Y. (2022). Cloud Detection Method Using CNN Based on Cascaded Feature Attention and Channel Attention. IEEE Trans. Geosci. Remote Sens..

[10] Zhang J., Wang Y., Wang H., Wu J., Li Y. (2022). CNN cloud detection algorithm based on channel and spatial attention and probabilistic upsampling for remote sensing image. IEEE Trans. Geosci. Remote Sens..

[11] Kaur Buttar P., Sachan M.K. (2022). Semantic segmentation of clouds in satellite images based on U-Net++ architecture and attention mechanism. Expert Syst. Appl..

[12] Du W., Fan Z., Yan Y., Yu R., Liu J. (2024). AFMUNet: Attention Feature Fusion Network Based on a U-Shaped Structure for Cloud and Cloud Shadow Detection. Remote Sens..

[13] Zhang G., Gao X., Yang Y., Wang M., Ran S. (2021). Controllably Deep Supervision and Multi-Scale Feature Fusion Network for Cloud and Snow Detection Based on Medium- and High-Resolution Imagery Dataset. Remote Sens..

[14] Wang W., Shi Z. (2022). An All-Scale Feature Fusion Network with Boundary Point Prediction for Cloud Detection. IEEE Geosci. Remote Sens. Lett..

[15] Li X., Yang X., Li X., Lu S., Ye Y., Ban Y. (2022). GCDB-UNet: A novel robust cloud detection approach for remote sensing images. Knowl. Based Syst..

[16] Wu K., Xu Z., Lyu X., Ren P. (2022). Cloud detection with boundary nets. ISPRS J. Photogramm. Remote Sens..

[17] Zhang C., Weng L., Ding L., Xia M., Lin H. (2023). CRSNet: Cloud and Cloud Shadow Refinement Segmentation Networks for Remote Sensing Imagery. Remote Sens..

[18] Zhao C., Zhang X., Kuang N., Luo H., Zhong S., Fan J. (2023). Boundary-aware bilateral fusion network for cloud detection. IEEE Trans. Geosci. Remote Sens..

[19] Du X., Wu H. (2023). Gated aggregation network for cloud detection in remote sensing image. Vis. Comput..

[20] Dong J., Wang Y., Yang Y., Yang M., Chen J. (2024). MCDNet: Multilevel cloud detection network for remote sensing images based on dual-perspective change-guided and multi-scale feature fusion. Int. J. Appl. Earth Obs..

[21] Li T., Wu D., Wang L., Yu X. (2022). Recognition algorithm for deep convective clouds based on FY4A. Neural Comput. Applic..

[22] Wang X., Iwabuchi H., Yamashita T. (2022). Cloud identification and property retrieval from Himawari-8 infrared measurements via a deep neural network. Remote Sens. Environ..

[23] Cao Y., Sui B., Zhang S., Qin H. (2023). Cloud Detection from High-Resolution Remote Sensing Images Based on Convolutional Neural Networks with Geographic Features and Contextual Information. IEEE Geosci. Remote Sens. Lett..

[24] Zhang J., Shi X., Wu J., Song L., Li Y. (2023). Cloud Detection Method Based on Spatial–Spectral Features and Encoder–Decoder Feature Fusion. IEEE Trans. Geosci. Remote Sens..

[25] Wang Y., Gu L., Li X., Gao F., Jiang T. (2023). Coexisting Cloud and Snow Detection Based on a Hybrid Features Network Applied to Remote Sensing Images. IEEE Trans. Geosci. Remote Sens..

[26] Dosovitskiy A., Beyer L., Kolesnikov A., Weissenborn D., Zhai X., Unterthiner T., Dehghani M., Minderer M., Heigold G., Gelly S. (2020). An Image is Worth 16×16 Words: Transformers for Image Recognition at Scale. arXiv.

[27] Liu Z., Lin Y., Cao Y., Hu H., Wei Y., Zhang Z., Lin S., Guo B. Swin transformer: Hierarchical vision transformer using shifted windows. Proceedings of the IEEE/CVF International Conference on Computer Vision.

[28] Li J., Wang Q. (2024). CSDFormer: A cloud and shadow detection method for landsat images based on transformer. Int. J. Appl. Earth Obs..

[29] Lu C., Xia M., Qian M., Chen B. (2022). Dual-Branch Network for Cloud and Cloud Shadow Segmentation. IEEE Trans. Geosci. Remote Sens..

[30] Gong C., Long T., Yin R., Jiao W., Wang G. (2023). A Hybrid Algorithm with Swin Transformer and Convolution for Cloud Detection. Remote Sens..

[31] Ma N., Sun L., He Y., Zhou C., Dong C. (2023). CNN-TransNet: A Hybrid CNN-Transformer Network With Differential Feature Enhancement for Cloud Detection. IEEE Geosci. Remote Sens. Lett..

[32] Gu G., Wang Z., Weng L., Lin H., Zhao Z., Zhao L. (2024). Attention Guide Axial Sharing Mixed Attention (AGASMA) Network for Cloud Segmentation and Cloud Shadow Segmentation. Remote Sens..

[33] Ge W., Yang X., Jiang R., Shao W., Zhang L. (2024). CD-CTFM: A lightweight CNN-transformer network for remote sensing cloud detection fusing multiscale features. IEEE J. Sel. Top. Appl. Earth Obs. Remote Sens..

[34] Gu H., Gu G., Liu Y., Lin H., Xu Y. (2024). Multi-Branch Attention Fusion Network for Cloud and Cloud Shadow Segmentation. Remote Sens..

[35] Gu G., Weng L., Xia M., Hu K., Lin H. (2024). Multipath Multiscale Attention Network for Cloud and Cloud Shadow Segmentation. IEEE Trans. Geosci. Remote Sens..

[36] Xu K., Wang W., Deng X., Wang A., Wu B., Jia Z. (2024). Transganet: Integration transformer with gradient-aware feature aggregation for accurate cloud detection in remote sensing imagery. IEEE Geosci. Remote Sens. Lett..

[37] Gao L., Li L., Yu J., Zhou X., Zou L., Fang N., Su X., Chen F. (2024). Swincloud: A hybrid network for cloud detection in thermal infrared remote sensing images. Int. J. Digit. Earth.

[38] Zhou W., Mo Y., Ou Q., Bai S. (2025). DFC-net: Dual-branch collaborative feature enhancement for cloud detection in remote sensing images. IEEE J. Sel. Top. Appl. Earth Obs. Remote Sens..

[39] Yu C., Wang J., Peng C., Gao C., Yu G., Sang N. Bisenet: Bilateral segmentation network for real-time semantic segmentation. Proceedings of the European Conference on Computer Vision (ECCV).

[40] Tang H., Li Z., Zhang D., He S., Tang J. (2025). Divide-and-Conquer: Confluent Triple-Flow Network for RGB-T Salient Object Detection. IEEE Trans. Pattern Anal. Mach. Intell..

[41] Li Z., Shen H., Liu Y. (2019). HRC_WHU: High-resolution cloud cover validation data. ISPRS J. Photogramm. Remote Sens..

